# Timing-controlled concept for extubation in brachycephalic dogs: α2–bridged on-demand extubation

**DOI:** 10.3389/fvets.2026.1762485

**Published:** 2026-02-24

**Authors:** Shotaro Nagahama

**Affiliations:** 1 JAVA Incorporated Association, Tokyo, Japan; 2 Department of Veterinary Anesthesia, Veterinary Teaching Hospital, Faculty of Applied Biological Sciences, Gifu University, Gifu, Japan

**Keywords:** atipamezole, brachycephalic dogs, Brachycephalicobstructive airway syndrome, extubation, recovery, upper-airway obstruction, volatile anesthetics, α2 adrenergic agonists

## Abstract

Brachycephalic dogs are overrepresented among peri and post-anesthetic respiratory complications, and many serious adverse events in small animals cluster around extubation and early recovery. A recurring clinical problem is a mismatch between apparent behavioral emergence and incomplete recovery of upper-airway stability, such that extubation may occur while residual anesthetic effect still depresses pharyngeal dilator activity and protective reflexes. Brachycephalic dogs have anatomically constrained, load-sensitive upper airways, making emergence a phase in which behavioral arousal may precede full recovery of airway stability. We propose a timing-controlled concept for extubation in brachycephalic dogs—α2–Bridged on-Demand Extubation (A2-ODE)—that decouples volatile washout from the timing of awakening and extubation. In A2-ODE, the vaporizer is turned off and washout is completed, as far as practicable, while the airway remains secured under a low to moderate-dose α2-agonist sedative bridge; awakening is then intentionally triggered by atipamezole immediately before planned extubation. The sequence is designed to avoid extubation within a volatile-associated vulnerable emergence range, stabilize the emergence phenotype, and provide clinician-controlled timing of wakefulness and extubation. We outline a stepwise protocol, discuss key prerequisites, limitations and hemodynamic considerations, and propose testable predictions for prospective clinical and physiologic studies in brachycephalic patients. This is a conceptual, hypothesis-generating article; A2-ODE is intended as a framework for future clinical studies rather than a validated protocol for routine use.

## 1 Introduction

Brachycephalic dogs have a disproportionate risk of peri and post-anesthetic respiratory complications compared with non-brachycephalic dogs (1), and in small animals many serious adverse events cluster around extubation and early recovery period (2). Even after corrective surgery for brachycephalic obstructive airway syndrome (BOAS), clinically important postoperative respiratory events remain frequent (3). These events likely reflect the combination of fixed anatomic narrowing and state-dependent loss of upper-airway tone characteristic of BOAS (4).

Commonly recommended peri-extubation strategies in veterinary practice—such as delaying extubation until gag and swallowing reflexes appear strong, maintaining sternal or elevated-head positioning, providing supplemental oxygen (e.g., facemask or flow-by), and using small doses of sedatives to blunt agitation—are widely used measures intended to support oxygenation and upper-airway patency in brachycephalic dogs (4, 5). However, these approaches do not directly control the relationship between residual anesthetic effect, apparent wakefulness, and recovery of upper-airway stability. Residual general anesthetic, whether inhalant or injectable, can create a concentration range in which eye opening, purposeful movement or agitation are present while pharyngeal dilator activity and protective reflexes remain impaired. In dogs—particularly brachycephalic breeds—prospective and retrospective studies instead document a high incidence of peri and post-anesthetic respiratory complications, hypoxemia and even re-intubation around the extubation period, indicating that emergence is likewise a clinically vulnerable phase in which apparent wakefulness does not guarantee airway safety (1, 3, 5, 6). Moreover, minimum alveolar concentration (MAC)-awake in dogs appears to represent a relatively large fraction of MAC compared with human data (7), suggesting that a clinically important “sub-MAC hazard zone” may be particularly prominent in canine practice, even though its precise extent has not been defined.

We propose a timing-controlled extubation concept for brachycephalic dogs—α2–Bridged On-Demand Extubation (A2-ODE). In A2-ODE, an α2-agonist is used to establish a calm, tube-tolerant state, volatile anesthetic is washed out to an end-tidal concentration of 0.0 while the airway remains secured, and emergence is then initiated at a deliberately chosen time point. In its index implementation, A2-ODE uses atipamezole to trigger rapid awakening immediately before planned extubation, with the intent of minimizing residual volatile and α2-mediated effects at the moment of airway testing. Extubation without atipamezole—whether after spontaneous recovery or under ongoing α2-based sedation—also conceptually fits within the same timing-controlled framework, but these non-reversal variants are not the primary focus of this article. This is a hypothesis-generating concept paper: A2-ODE is presented as a testable timing-controlled extubation strategy rather than a fully validated protocol, and its benefits and risks require evaluation in prospective controlled clinical studies in brachycephalic dogs.

## 2 Pathophysiological and anesthetic rationale

### 2.1 Brachycephalic airway and upper-airway tone

BOAS results from marked maxillary shortening without a proportional reduction in nasal and pharyngeal soft tissues, creating diffuse “soft-tissue crowding” of the upper-airway that is often lifelong and progressive (4). Primary changes such as stenotic nares, an overlong or thickened soft palate, macroglossia and aberrant nasal turbinates increase upper-airway resistance (4), while chronic inspiratory effort and negative intraluminal pressure promote secondary lesions including everted laryngeal saccules and laryngeal collapse (8, 9).

Imaging and endoscopic studies identify the nasal cavity, nasopharynx and laryngopharynx as major sites of obstruction: many pugs and French bulldogs show intranasal mucosal contact points and hypertrophic conchae that markedly narrow the airflow pathway compared with mesocephalic controls (10, 11). Functional assessments using whole-body barometric plethysmography demonstrate chronically increased upper-airway resistance and altered inspiratory patterns, with flow–volume loop distortion and indices of inspiratory drive correlating with BOAS grade and conformational risk factors such as reduced muzzle length (12, 13).

Collectively, these data support the view that the brachycephalic upper-airway behaves as a load-sensitive, tone-dependent collapsible segment: even in the awake state, airflow must traverse a narrowed, tortuous conduit, repeatedly exposed to large inspiratory pressure swings, and it is plausible that relatively small decrements in neuromuscular drive to pharyngeal dilator muscles can precipitate disproportionate increases in resistance and dynamic collapse, particularly under conditions that reduce upper-airway tone such as sleep, sedation or general anesthesia (14, 15).

### 2.2 Residual volatile anesthetics and the “sub-MAC hazard zone”

Emergence from general anesthesia is a graded process in which different functions recover at different anesthetic concentrations. In dogs, sevoflurane MAC-awake appears relatively high as a fraction of MAC, suggesting that behavioral responsiveness can return at comparatively higher fractions of MAC than in humans (7). Clinically, peri-anesthetic studies in dogs show that serious adverse events cluster around the recovery period, and that brachycephalic breeds in particular are overrepresented among post-anesthetic respiratory complications, hypoxemia, and unplanned re-intubation (1–3). Taken together, these observations support the idea that in brachycephalic patients the emergence phase is a vulnerable window in which apparent wakefulness does not necessarily equate to safe upper-airway function.

From a mechanistic standpoint, detailed measurements of upper-airway mechanics during light planes of volatile anesthesia are sparse in dogs. However, canine MAC, MAC-awake, and MAC-extubation data indicate that these endpoints shift to lower end-tidal anesthetic concentrations when opioids, α2-agonists, or other central nervous system (CNS)-active drugs are co-administered (7, 16). Thus, for any given “low” end-tidal anesthetic concentration near emergence, the combined anesthetic state produced by residual inhalant plus adjunct drugs may still exert clinically relevant effects on arousal, ventilation, and airway protection.

Comparable vulnerable states may also arise with injectable hypnotics. Direct canine data linking low, residual propofol or alfaxalone concentrations to impaired airway patency are limited, but peri-anesthetic reviews in brachycephalic dogs describe severe upper-airway obstruction developing during pre-intubation sedation and induction, before the airway is secured (5). It is therefore clinically plausible that, during recovery from injectable protocols, dogs may also pass through phases in which movement or apparent wakefulness precede full restoration of upper-airway stability.

In this article, the term “sub-MAC hazard zone” is used as a shorthand for a range of sub-MAC volatile anesthetic concentrations around and below MAC-awake in dogs, in which overt signs of emergence (eye opening, purposeful movement, and agitation) may coexist with persistent drug effects on upper-airway tone, respiratory drive, and protective reflexes. The precise boundaries of this zone are protocol and patient-dependent and remain incompletely defined in dogs, but clinical outcome data in brachycephalic patients suggest that such a vulnerable emergence range is clinically important. From a pragmatic standpoint, inhalant-based maintenance offers an advantage for the present concept: end-tidal volatile anesthetic concentration provides a continuously visible surrogate for the washout of residual volatile influence during recovery. Low-solubility agents such as sevoflurane and desflurane are therefore particularly convenient, because their short decrement times allow the clinician to time A2-ODE relative to a reasonably predictable decline in volatile influence, whereas with total intravenous anesthesia comparable residual hypnotic effects may persist despite the absence of any measurable volatile concentration. Throughout this article, end-tidal volatile anesthetic concentration is used pragmatically as an indicator of declining residual volatile anesthetic influence during emergence; transient gradients between end-tidal and effect-site concentrations may exist during washout.

## 3 Pharmacologic rationale: α2-agonists and atipamezole

### 3.1 α2-agonist sedation during recovery

Within the A2-ODE concept, α2-agonists such as medetomidine and dexmedetomidine are used as a sedative bridge rather than as primary anesthetics. By stimulating central α2A-receptors in the locus coeruleus and related nuclei, they reduce noradrenergic output and produce a sleep-like, non–REM–resembling state that can be rapidly reversed by α2-antagonists (17, 18). In dogs, medetomidine and dexmedetomidine provide dose-dependent sedation, muscle relaxation and analgesia, and substantially reduce requirements for volatile anesthetics and injectable hypnotics (19, 20). Their cardiovascular profile—increased systemic vascular resistance with bradycardia and a consequent reduction in cardiac output—requires careful selection and monitoring (19). At clinically typical doses, direct respiratory depression is generally modest and spontaneous ventilation can often be maintained (17, 19). Randomized studies in dogs have reported improved recovery quality and reduced dysphoria/agitation with dexmedetomidine administered toward the end of inhalant anesthesia; in protocols without routine antagonism, extubation may be delayed because α2 effects are allowed to dissipate spontaneously (21–23).

In A2-ODE, these properties are applied in a specific way: after vaporizer shutoff, a low to moderate-dose α2-agonist is initiated or continued to maintain a calm, recumbent, spontaneously breathing state while volatile anesthetic concentrations decline toward zero. Non–α2-based analgesia (local anesthetic techniques, opioids, NSAIDs) is ensured during this period so that pain control will persist after reversal. This α2-agonist–mediated sedation maintenance phase functions as a bridge: it provides a defined, relatively stable state from which atipamezole can later trigger on-demand awakening.

### 3.2 On-demand reversal with atipamezole

Atipamezole is a selective α2-adrenergic antagonist developed to reverse medetomidine-induced sedation and analgesia in small animals. By competitively displacing α2-agonists from central and peripheral α2-receptors, it restores noradrenergic tone and sympathetic outflow (24). In dogs sedated with medetomidine, intramuscular or intravenous atipamezole produces dose-dependent reversal of sedation and α2-associated bradycardia within minutes; clinically used dose ratios are typically several-fold relative to the medetomidine dose on a μg/kg basis (24, 25). Pharmacokinetic studies further suggest that reversal reflects not only rapid receptor-level displacement but also physiologic changes that can increase α2-agonist clearance after antagonism (26). Analgesic as well as sedative effects are antagonized. In nociceptive withdrawal reflex studies, medetomidine or dexmedetomidine-induced increases in withdrawal threshold return toward baseline within minutes of atipamezole administration, in parallel with improvement in behavioral sedation scores (27). Electroencephalographic recordings in dexmedetomidine-infused dogs likewise show an abrupt transition from slow-wave, spindle-rich α2-sedation patterns to wake-like activity after atipamezole infusion, accompanied by rapid normalization of autonomic variables (28).

Within the A2-ODE framework, these properties enable an “on-demand” shift from a controlled α2-sedated bridge to a behaviorally awake state after volatile anesthetic has been largely eliminated; this transition is expected to facilitate return of airway protective reflexes and creates a clinician-controlled time point for attempting extubation.

### 3.3 Integrated rationale for α2-based bridging

Taken together, these properties explain why α2-agonists paired with atipamezole are proposed as the primary – and in practice sole – bridging drugs in the A2-ODE framework. During volatile washout, low to moderate-dose α2-agonist sedation can provide a stable, non-dysphoric state that is deep enough to permit safe maintenance of endotracheal intubation in brachycephalic dogs, while usually preserving spontaneous breathing and allowing careful physiological assessment (19, 20). The availability of a selective antagonist then allows this bridging sedation maintenance phase to be terminated rapidly and predictably, enabling the clinician to decouple the timeline of volatile elimination from the timing of awakening and extubation (24, 25).

In contrast, injectable hypnotics such as propofol or alfaxalone can readily produce a tube-tolerant state, but they lack a specific antagonist that would permit rapid, on-demand return of consciousness; emergence is therefore constrained by context-sensitive pharmacokinetics and an additional “tail” of hypnotic action that A2-ODE explicitly seeks to avoid.

Benzodiazepines and opioids do have antagonists (e.g., flumazenil, naloxone), but they still do not meet the practical requirements of A2-ODE bridging when used as primary sedatives. In dogs, benzodiazepine sedation can be inconsistent and may include paradoxical behavioral excitation; midazolam-induced agitation and abnormal behaviors were documented in healthy dogs and were not reliably prevented by co-administration of acepromazine or methadone, underscoring the risk that a predictable, calm, tube-tolerant bridge may be difficult to achieve with benzodiazepines alone (29).

Opioids are primarily analgesics and can produce sedation, but in dogs the recovery phenotype is not consistently dose-predictable, and adverse effects such as nausea, vomiting, ptyalism, and increased panting can complicate efforts to maintain a quiet, stable intubated state and to interpret respiratory effort (30). Moreover, antagonizing opioids purely to “time” awakening would simultaneously remove a desired analgesic component and risks contributing to dysphoric recoveries, which runs counter to the intent of reaching extubation with stable comfort and minimal pharmacologic tails.

Accordingly, within the A2-ODE framework, α_2_-agonist sedation with atipamezole reversal is treated as the bridging sedation maintenance phase, and other sedative–hypnotic classes are not relied upon to maintain intubation during washout. Non-α2 analgesic components (local or regional anesthetic techniques, opioids, NSAIDs) are still used, but in their intended role as analgesic foundations rather than as timing-critical bridge drugs.

## 4 Proposed concept: α2–bridged on-demand extubation (A2-ODE)

**Figure 1:**
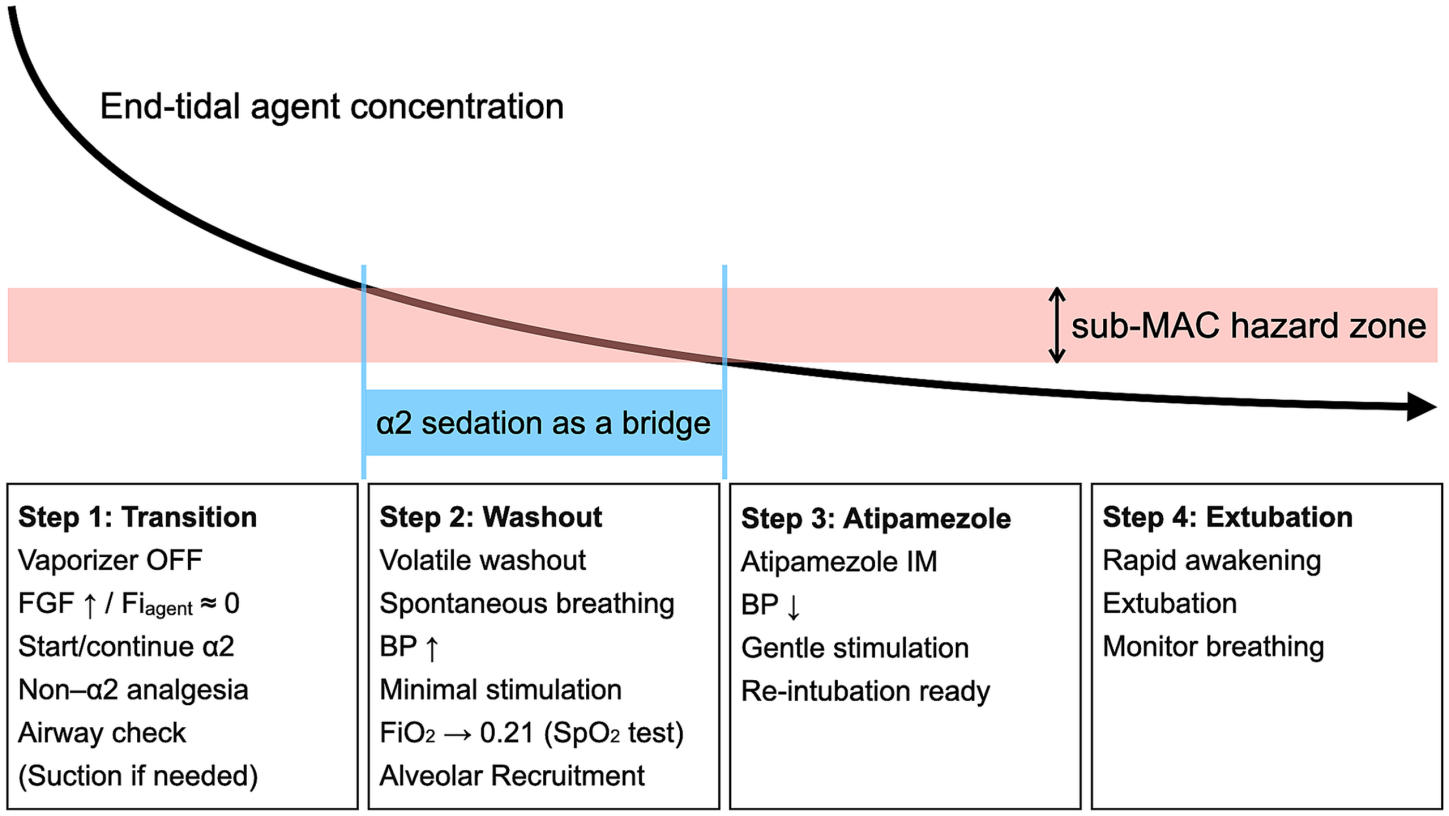
Timing-controlled concept of α2–bridged on-demand extubation (A2-ODE). A2-ODE separates volatile washout from the moment of awakening and extubation by maintaining intubation under an α2-agonist bridge during washout and readiness assessment, and then initiating rapid emergence via atipamezole immediately before planned extubation. The intent is to complete washout as far as practicable while the airway remains secured, and to align extubation with the early post-antagonism period when residual volatile hypnotic effect is minimized. Monitoring and rescue readiness are maintained throughout. The declining curve denotes end-tidal agent concentration, used pragmatically as an indicator of declining residual volatile anesthetic influence during recovery (schematic, not quantitative). FGF, fresh gas flow; Fi_agent_, inspired agent fraction; BP, blood pressure.

The following workflow is a timing-controlled recovery concept derived from the rationale presented in this article and the authors’ clinical experience. It is offered as a conceptual template rather than a fixed protocol and should be adapted to local practice, equipment, and patient comorbidities (Figure 1). A clinical checklist summarizing the A2-ODE workflow and rescue readiness is provided as Supplementary Material S1.

In A2-ODE, volatile washout is conducted while the airway remains secured under an α2-agonist bridge, and awakening is then deliberately triggered with atipamezole immediately before planned extubation, with standard monitoring maintained throughout. The concept is most straightforward under inhalant maintenance because end-tidal agent concentration provides an accessible indicator of declining residual volatile anesthetic influence during emergence; additional sedative–hypnotic “tails” are avoided where possible to preserve interpretability of readiness assessment and timing control.

### 4.1 Step 1—transition from maintenance anesthesia and establishment of α2-agonist sedation

When the procedure requiring general anesthesia is complete and no further noxious stimulation is anticipated, the volatile anesthetic is discontinued by turning off the vaporizer, and fresh gas flow is increased to reduce the inspired fraction of volatile anesthetic as close to zero as practicable, particularly when a circle system is used. The immediate aim is to eliminate ongoing circuit delivery so that subsequent changes reflect washout from the lungs and tissues rather than persistent low-level exposure with each breath.

If anesthesia has been maintained with controlled or assisted ventilation, ventilatory support is transitioned toward spontaneous breathing when feasible while maintaining continuous capnography and oxygenation monitoring; if spontaneous breathing has been present throughout, the existing pattern is supported and monitored during the transition.

During the same period, α2-agonist sedation is established or maintained as a bridging sedation maintenance phase. In most cases, an initial dexmedetomidine dose of approximately 0.5 μg/kg IV, administered as one or several small boluses and titrated to effect, is sufficient to achieve a calm, recumbent, tube-tolerant state while preserving spontaneous breathing and interpretable respiratory patterns. Intravenous administration is emphasized here because IV access is routinely available under general anesthesia and allows fine titration; however, the conceptual framework of A2-ODE does not depend on a specific route, and equivalent regimens may be adapted to local protocols where IV titration is not practical. Example starting doses are summarized in Table 1.

**Table 1:** Key Points for α2–bridged on-demand extubation (A2-ODE).

Domain	Key points
Case selection	Consider airway risk (BOAS severity, prior recovery events) versus hemodynamic tolerance for an α2 bridge (afterload-sensitive disease, marked brady arrhythmia risk).
Analgesia prerequisite	Ensure multimodal non–α2 analgesia is in place before any atipamezole use (local anesthetic techniques, opioids, NSAIDs as appropriate).
Concomitant drug “tails”	Prefer low/predictable sedative–hypnotic tails at reversal; avoid adding non–α2 sedatives during washout unless clinically necessary, as they can obscure readiness assessment and weaken timing control.
Airway check and clearance	Complete any potentially stimulating airway manipulation (oral/pharyngeal inspection, suction as needed) while the airway remains secured; aim for minimal stimulation during bridged washout.
Washout readiness	ET_agent_ 0.0%; ventilation and oxygenation within general peri-anesthetic targets (e.g., SpO₂ ≥ 95% without sustained hypercapnia); hemodynamics acceptable; rescue plan and readiness to re-secure the airway confirmed.
Extubation approach	Atipamezole-triggered emergence after volatile elimination, followed by prompt extubation once airway control returns. Possible non-reversal variants (not the primary focus of this article) include completing volatile washout under α2-based sedation and either extubating sedated or allowing full spontaneous emergence without atipamezole, while preserving the principle of avoiding extubation during volatile-dominated light planes.
Hemodynamics around reversal	Potential rapid transition from α2-associated hypertension to post-reversal hypotension around atipamezole.
Post-extubation vigilance	Close observation for obstruction/hypoventilation; SpO_2_ monitoring; oxygen and airway rescue immediately available until recovery criteria are met.
Example starting doses (provisional)	A practical starting option for A2-ODE bridging is dexmedetomidine 0.5 μg/kg IV, with small additional IV doses administered if sedation is judged insufficient while cardiovascular status remains acceptable. For reversal, atipamezole 5 μg/kg IM can be used as an initial dose, with further IM increments titrated to effect if awakening is inadequate. These values are intended as illustrative starting points, derived from commonly used clinical protocols and published dose ranges for dexmedetomidine/atipamezole in dogs (19, 20, 24), and must be adapted to local safety checks, monitoring standards, and individual cardiovascular risk.

BOAS, brachycephalic obstructive airway syndrome; ET_agent_, end-tidal agent concentration.

Non–α2-based multimodal analgesia is ensured so that analgesia will persist after α2 antagonism. Before proceeding to the next step, any potentially stimulating airway manipulation (inspection and gentle suctioning as needed) is completed while the airway remains secured, so that the subsequent bridged washout phase can be conducted with minimal stimulation.

### 4.2 Step 2—bridged washout and pre-reversal readiness

A fixed “washout time” cannot be prescribed for all cases. Even with normal ventilation and cardiac output, the decline of volatile anesthetic effect is context-sensitive: peripheral uptake and back-diffusion vary with agent solubility, anesthetic duration, body composition, and physiologic conditions, such that the low-concentration “tail” can differ markedly between patients. Accordingly, A2-ODE operationalizes washout by targeting inspired agent fraction ≈ 0 and using end-tidal agent concentration (and its stability over time) together with cardiorespiratory readiness criteria, rather than relying on a universal number of minutes.

During this phase, the dog remains intubated and sedated under the α2-agonist while volatile anesthetic is washed out and readiness for reversal is optimized. Fresh gas flow is set to minimize rebreathing and maintain inspired agent fraction ≈ 0, and FiO_2_ is adjusted as needed. Where feasible, FiO_2_ may be reduced toward an air-equivalent level (≈ 0.21) while SpO_2_ is monitored to assess oxygenation without the masking effect of high inspired oxygen. The physiologic targets during A2-ODE do not differ from general peri-anesthetic goals; in most dogs, a practical aim is to maintain SpO_2_ in the mid to high-90s (e.g., ≥ 95%), with persistent values below this range prompting correction before proceeding and hypoxemia (e.g., SpO_2_ < 95%) considered unacceptable for planned reversal and extubation.

Ventilatory support is provided only as needed to maintain a stable respiratory pattern and acceptable gas exchange; when spontaneous breathing is adequate, no assistance is required. Capnography and respiratory effort are monitored continuously. Sustained hypercapnia is not accepted as a baseline for atipamezole reversal and planned extubation; if CO₂ levels remain consistently elevated, ventilation should be optimized and a clear improvement in gas exchange established before proceeding. If additional objective assessment of ventilation or oxygenation is considered useful, arterial or venous blood gas analysis can be performed before reversal and extubation. External stimulation is minimized during bridged washout; upper-airway manipulation (e.g., suctioning) is avoided unless clinically necessary (e.g., accumulating secretions, regurgitation, bleeding). The dog is typically maintained in sternal recumbency with easy oral access.

This phase ends once the end-tidal volatile anesthetic concentration has reached 0.0 and the predefined readiness criteria listed above for ventilation, oxygenation, hemodynamics, airway condition, and analgesia have been met.

### 4.3 Step 3—administration of atipamezole and supported awakening

Once end-tidal volatile anesthetic concentration has reached 0.0% and cardiorespiratory/airway readiness criteria have been met, atipamezole is administered intramuscularly to antagonize the α2-agonist. A practical starting dose is approximately 5 μg/kg IM, with titration according to clinical response and local protocol; this range is intended as provisional guidance and should be adapted to institutional practice and monitoring standards (Table 1).

There is no fixed interval between establishing α2-agonist sedation and atipamezole reversal in A2-ODE. The α2-agonist is maintained at a steady sedative level during the bridged washout phase, and reversal is deferred until end-tidal anesthetic concentration has reached 0.0% and remains at that level on successive measurements, and cardiorespiratory readiness criteria are met; antagonizing too early effectively bypasses the bridge, whereas delaying reversal once these criteria are satisfied mainly prolongs recovery without clear additional safety benefit.

Because reversal can convert a stable α2-sedated state into wakefulness over a short time scale, the dog is physically supported and the environment is controlled. Abrupt noises and rough handling are avoided. At the same time, the patient is not left completely unstimulated: gentle, continuous tactile and verbal stimulation after atipamezole can help promote a predictable transition to wakefulness and may reduce the likelihood of a delayed, startle-triggered “explosive” rise from a sleep-like state. Monitoring of consciousness level, ventilation and hemodynamics continues without interruption, and equipment for immediate airway intervention remains ready.

Within the A2-ODE framework, early behavioral arousal while end-tidal volatile anesthetic concentration remains above 0.0% is not a cue to proceed to extubation but rather a prompt to reinforce the α2-agonist bridge (e.g., with small titrated increments) and continue the bridged phase, so that extubation is avoided while clinically meaningful volatile influence is still likely.

### 4.4 Step 4—prompt extubation and immediate post-extubation care

Once atipamezole is administered after end-tidal volatile anesthetic concentration has reached 0.0% and remained stable, awakening is typically brisk. Because volatile anesthetic influence has been largely eliminated by design, the early post-reversal period is usually characterized by relatively clear wakefulness with limited ataxia rather than a prolonged, dysphoric intermediate phase. When consistent signs of airway control and purposeful wakefulness are present—such as coordinated head lifting, increased jaw tone, and reproducible swallowing and/or coughing—the endotracheal tube is removed promptly in a controlled fashion while maintaining the chosen body position and manual support.

Immediately after extubation, respiratory pattern, effort and upper-airway sounds are assessed, and oxygenation is monitored (SpO_2_). Supplemental oxygen (facemask or flow-by) is provided as needed according to local practice. Close observation continues for signs of upper-airway obstruction, hypoventilation, agitation or regurgitation. Equipment and drugs for airway rescue remain immediately available, including agents for rapid re-induction (e.g., propofol or alfaxalone) and locally standard devices for re-securing the airway. Monitoring and support are maintained until predefined recovery criteria are met, such as stable spontaneous breathing with acceptable respiratory pattern and effort, SpO_2_ ≥ 95% on room air or low-flow oxygen, and absence of clinically relevant airway distress.

If clinically significant upper-airway obstruction or hypoventilation persists despite brief supportive measures, the event should be treated as a failed extubation attempt and airway control re-established (e.g., oxygen supplementation with assisted ventilation and prompt re-securing of the airway) rather than allowing prolonged obstructed breathing. Within the A2-ODE concept, if upper-airway obstruction after extubation appears substantially worse than the pre-anesthetic baseline, this raises concern that the airway is either still influenced by residual anesthetic or other CNS-active drugs (insufficient washout) or has been newly compromised, for example by procedure-related oedema.

## 5 Discussion

### 5.1 Limitations, risks and scope of application

Several limitations and potential risks of the A2-ODE approach deserve consideration (Table 1).

*Cardiovascular effects and case selection*: α2-agonists can produce increased systemic vascular resistance and bradycardia, leading to reductions in cardiac output, particularly at higher doses (19, 20). Accordingly, A2-ODE requires case-by-case risk–benefit assessment and may be unsuitable in dogs with conduction disturbances, severe cardiac disease, markedly reduced cardiac reserve, or afterload-sensitive lesions (e.g., clinically significant mitral regurgitation). In dogs with known or suspected cardiovascular compromise, the potential benefits of a timing-controlled extubation strategy must be weighed against the hemodynamic liabilities of α2-agonist use; in some such patients, a more conventional emergence without an α2-based bridge may be preferable. Evidence from clinical studies and a systematic review suggests that dexmedetomidine-based protocols can be used under careful monitoring and typically yield lower heart rate and higher arterial pressure than protocols without α2-agonists (31, 32). Because A2-ODE includes planned antagonism, clinicians should also anticipate potentially rapid blood pressure transitions around the time of atipamezole administration and maintain continuous blood pressure monitoring and readiness to support hemodynamics during the reversal–extubation window. Within the A2-ODE framework, there is no protocol-specific “maximum” α2-agonist dose; α2-agonists are kept within the same low-dose sedative range that the clinician would ordinarily regard as acceptable for that individual patient, and if satisfactory conditions for bridging cannot be achieved within that range, the case is better considered unsuitable for A2-ODE than an indication to escalate the α2 dose further.

*Regurgitation, vomiting and aspiration*: Brachycephalic dogs are at increased risk of peri-anesthetic regurgitation, vomiting and aspiration, irrespective of the specific recovery strategy. A2-ODE is not expected to eliminate these risks or uniquely amplify them compared with other α2 and opioid-based protocols, because it uses the same emetogenic drug classes and primarily reorganizes the timing of volatile washout and extubation while maintaining a secured airway during much of the high-risk period. In higher-risk cases, clinicians should apply the same regurgitation/aspiration precautions that they would use for α2-based anesthesia in brachycephalic dogs in general (including local policies on gastric management and antiemetic use) and ensure readiness to re-secure the airway if clinically indicated.

*Reversal-triggered versus non-reversal implementations*: In this article, the index implementation of A2-ODE uses atipamezole-triggered emergence to concentrate extubation into a brief period after volatile and α2-mediated effects have largely resolved. Conceptually, the same timing-controlled framework can be applied without pharmacologic reversal in two ways. In one variant, extubation is performed under ongoing α2-based sedation once volatile washout is complete and airway control is judged adequate, with residual α2 effect accepted as part of the immediate post-extubation state. In another variant, both volatile and α2 effects are allowed to dissipate and extubation occurs after spontaneous recovery, provided that the principle of completing volatile washout before extubation is maintained. Which implementation is preferred in a given case will depend on patient factors, clinician experience, and local risk tolerance. The present description focuses on the reversal-triggered variant because it most directly tests the hypothesis that concentrating emergence into a short, well-defined interval after volatile elimination can reduce exposure to sub-MAC volatile states at the moment of extubation.

*Analgesia must be secured before reversal*: Atipamezole reverses not only sedation but also *α*2-mediated analgesia; experimental data indicate that nociceptive thresholds return toward baseline within minutes of antagonism (27). Therefore, multimodal non–*α*2 analgesic components (local anesthetic techniques, opioids, NSAIDs) should be established before reversal to avoid pain-driven agitation and increased respiratory effort at a vulnerable time. As with any peri-anesthetic protocol that relies on *μ*-opioid agonists, residual opioid effect may contribute to dysphoria or panting during recovery in some dogs; this possibility is not specific to A2-ODE but should be considered when interpreting emergence behavior and planning rescue sedation.

*Interactions with other CNS-active drugs*: Because atipamezole antagonizes only the α2-agonist component, residual effects of concurrent non–*α*2 drugs can dominate the immediate recovery phenotype. This is most relevant when ketamine is part of the protocol, where emergence quality may be variable and can become more apparent after α2 reversal (33). Benzodiazepines may also produce unreliable sedation with paradoxical excitation in some dogs (29). Accordingly, when antagonism is used as a deliberate wake-up trigger, A2-ODE favors minimizing unpredictable sedative–hypnotic “tails” at the time of reversal.

*Applicability beyond inhalant-based maintenance*: A2-ODE is formulated primarily for inhalant maintenance, where end-tidal agent concentration provides a practical, continuously observable indicator of declining residual volatile anesthetic influence during emergence. While the sequence can be applied with any volatile agent, lower-solubility agents (e.g., sevoflurane, desflurane) are convenient because decrement times are short (34, 35). Extending the framework to TIVA (e.g., propofol or alfaxalone infusions) would require alternative markers or endpoints, because an end-tidal concentration surrogate is not available and recovery is governed by context-sensitive declines in drug concentrations and effects (36). Although plasma drug concentrations would in principle be the most direct descriptor of residual effect, they cannot be measured in real time at the bedside in current veterinary (or human) practice, because available assays require intermittent sampling and off-site analysis. Accordingly, the present concept is limited to implementations that can be guided by widely accessible clinical and monitoring variables rather than by blood-level endpoints.

*Evidence base and extrapolation*: The mechanistic rationale for A2-ODE combines canine data with a limited degree of extrapolation from human anesthesia studies. In brachycephalic dogs, prospective and retrospective veterinary studies document increased rates of peri and post-anesthetic respiratory complications and recovery-phase hypoxemia, particularly around extubation, supporting the clinical relevance of the emergence window (1, 3, 37). Canine data also describe MAC, MAC-awake and MAC-extubation behavior and their modification by adjunct drugs (7, 16). By contrast, detailed measurements of upper-airway collapsibility and dilator muscle behavior during light volatile or propofol anesthesia come mainly from human volunteer studies (36, 38, 39) and are cited here only as mechanistic analogues rather than as species-equivalent evidence. Overall, species-specific risk and applicability in this article are grounded in veterinary outcome data, and the A2-ODE concept requires prospective controlled clinical evaluation in dogs.

*Broader applicability beyond brachycephalic conformation*: Although brachycephalic dogs are a clinically important target group, the core risk addressed by A2-ODE—state-dependent upper-airway obstruction during emergence—may also be present in non-brachycephalic dogs in whom clinically meaningful obstruction is unmasked during pre-intubation sedation or induction. In such cases, A2-ODE may be considered as a recovery workflow option, subject to the same hemodynamic and staffing constraints.

*Monitoring and staffing requirements*: Implementation of A2-ODE presupposes at least the monitoring and staffing standards expected for major inhalant anesthesia in brachycephalic dogs. Continuous ECG, capnography, pulse oximetry, and non-invasive blood pressure monitoring should be maintained throughout the bridged washout and reversal–extubation window, with invasive arterial pressure monitoring strongly recommended when available. A trained anesthetist (or anesthesia-trained veterinarian/nurse) should be continuously present at the bedside during atipamezole administration and the immediate post-extubation period, with an additional assistant available for patient positioning, manual support, and rapid airway rescue if required.

*Operational considerations and evidence gap*: Prospective studies evaluating A2-ODE as a structured recovery workflow in brachycephalic dogs are not yet available; therefore, procedural elements (e.g., handling/stimulation and oxygen titration) should be regarded as experience-based until evaluated in prospective clinical studies or controlled clinical trials.

### 5.2 Future research and testable predictions

The A2-ODE framework is readily testable in clinical and physiologic studies. A pilot prospective trial could compare A2-ODE with standard recovery management in brachycephalic dogs using peri and post-extubation hypoxemia, overt obstructive episodes, unplanned re-intubation, and validated recovery-quality scores as core endpoints. Secondary outcomes could include time to extubation, time to sternal recumbency, and the need for rescue sedation or analgesia, with stratification by BOAS grade to identify subgroups most likely to benefit.

Mechanistic studies could focus on airway mechanics and gas exchange across key phases of the sequence, for example by measuring esophageal and nasopharyngeal pressures or estimating pharyngeal critical closing pressure before and after atipamezole. Parameter-optimization work could then examine how α2-agonist dose, timing, and route, as well as atipamezole dosing strategies, trade off hemodynamic stability against the sharpness of awakening. Variants—including lower-dose *α*2 strategies, partial or staged reversal, integration with staged extubation techniques, or omission of atipamezole after washout when continued α2 sedation is desired—can be evaluated within the same conceptual framework to define the practical envelope of the approach.

A pragmatic prediction is that upper-airway obstruction observed during pre-intubation sedation/induction will identify patients at higher risk of peri-extubation obstruction and unplanned airway interventions, enabling simple peri-anesthetic risk stratification regardless of head conformation.

Beyond structurally high-risk airways, future work could also explore whether the same timing-controlled, antagonist-triggered framework mitigates other recovery-phase problems linked to residual low-dose volatile or injectable anesthetic effects (such as dysphoric or unstable emergence in non-brachycephalic dogs). Such extensions would, however, fall outside the primary scope of the present concept and would require indication-specific prospective validation.

### 5.3 Conclusion

The A2-ODE concept integrates three strands of evidence. First, brachycephalic dogs have structurally constrained, load-sensitive upper airways due to their underlying conformation, which makes them inherently more susceptible to upper-airway obstruction when neuromuscular tone is reduced. This baseline vulnerability is anatomical rather than pharmacologic; anesthetic drugs modulate how it is expressed during emergence but do not create it. Second, residual anesthetic effect can create vulnerable emergence states in which behavioral arousal precedes full recovery of upper-airway stability—described for inhalant agents as a sub-MAC hazard zone. Third, *α*2-agonists paired with atipamezole allow recovery to be reshaped into a defined sedation maintenance phase followed by rapid antagonism. This enables deliberate control of extubation timing.

By organizing recovery around these elements, A2-ODE reframes extubation in brachycephalic dogs as a problem of timing and state control superimposed on a fixed conformational risk, rather than as a passive by-product of volatile washout. Whether this reframing leads to fewer respiratory complications, smoother recoveries, or more predictable extubation is an empirical question. A2-ODE is presented as a mechanistically coherent, testable hypothesis and a procedural template—derived from available evidence and clinical experience—that can be evaluated, refined, or refuted through prospective clinical and physiologic studies.
