# Supplementary material for: Timing-controlled concept for extubation in brachycephalic dogs: α2–bridged on-demand extubation
Source: Front Vet Sci. 2026 Feb 24;13:1762485. doi: 10.3389/fvets.2026.1762485 (PMC12974264; doi:10.3389/fvets.2026.1762485)
Supplement: Supplementary file 1 [file Supplementary_file_1.pdf]

frontiers

Supplementary Material 1. A2-ODE workflow checklist

This checklist summarizes key steps for α2-bridged on-demand extubation (A2-ODE), primarily in brachycephalic dogs at increased risk of peri-anesthetic upper-airway obstruction. Principles may be adapted to other high-airway-risk patients.

1. Case selection / exclusions

☐ General anesthesia with supervised recovery planned

☐ No major contraindication to α2-agonist use (e.g. severe cardiac failure, marked conduction disturbance, extreme afterload sensitivity)

☐ No pre-existing decision against α2-based recovery strategy

☐ Owner consent obtained for general anesthesia and proposed recovery plan

2. Intra-anesthetic prerequisites

☐ Standard monitoring in place (ECG, capnography, SpO_2, BP) and reliable IV access secured

☐ Multimodal non-α2 analgesia ensured (opioids, local/regional techniques, NSAIDs as appropriate)

☐ Surgical / diagnostic procedure completed; no further major noxious stimulus expected

3. Step 1 – Transition from maintenance and establish α2 sedation

☐ Vaporizer turned off; fresh gas flow set to reduce Fi_{agent} toward 0

☐ Ventilation transitioned toward spontaneous breathing when feasible, with capnography and SpO_2 monitored

☐ Dexmedetomidine titrated IV to a calm, recumbent, tube-tolerant state (e.g. starting around 0.5 µg/kg IV, adjusted to response and local protocol)

☐ Airway inspected and obvious secretions/fluids removed while still intubated

☐ Airway rescue equipment and re-induction drugs available at the bedside

4. Step 2 – Bridged washout and readiness check

☐ $Fi_{agent} \approx 0$

☐ SpO2 ≥ 95 % maintained without sustained desaturation

☐ $EtCO_2$ within acceptable range

☐ Blood pressure acceptable for the individual; no progressive hypotension or severe hypertension

☐ No new concern about airway obstruction while intubated

☐ Staff, monitoring, and environment ready for reversal and planned extubation attempt

5. Step 3 – Atipamezole and supported awakening

☐ Atipamezole prepared for IM use (e.g. starting around 5 µg/kg IM, adjusted to α2-agonist dose, timing, and local protocol)

☐ Atipamezole administered when ET_{agent} is 0.0 % and Step-2 criteria are met

☐ An experienced anesthesia team member remains continuously at the bedside after reversal

☐ Gentle handling and verbal contact used to support a controlled transition to wakefulness

☐ Airway rescue equipment and re-induction agents immediately available

6. Step 4 – Extubation and immediate post-extubation care

☐ Consistent signs of airway control and purposeful wakefulness present (e.g. coordinated head lift, increased jaw tone, reproducible swallowing/coughing)

☐ Endotracheal tube removed promptly in a controlled manner with manual support and appropriate positioning

☐ Post-extubation: respiratory pattern, effort, and upper-airway sounds re-assessed; SpO_2 monitored

☐ Supplemental oxygen provided as needed (facemask or flow-by) and observation continued for early signs of obstruction, hypoventilation, agitation, or regurgitation

☐ If clinically significant upper-airway obstruction or hypoventilation persists despite brief supportive measures, the episode is treated as a failed extubation attempt and airway control is re-established without delay (e.g. assisted ventilation and re-securing of the airway using the locally preferred technique)
